# Assessing AI-generated smoking cessation advice for patient education in primary care

**DOI:** 10.1186/s12875-026-03360-z

**Published:** 2026-05-18

**Authors:** Canan Tuz, Demet Merder, Bahar Urun Unal, Fethi Sada Zekey, Kubra Uyar Zekey, Tuncay Muge Alvur

**Affiliations:** 1 Family Medicine Department, Kocaeli University Medical School, Umuttepe, Kocaeli, Turkey; 2 Family Medicine Department, Health Sciences University, Izmir Tepecik Health Practice And Research Center, Konak, İzmi̇r, Turkey; 3 Family Medicine Department, Medical School, Selçuk University, Selçuk, Konya, Turkey; 4 Family Medicine Department, Bozok University Medical School, Yozgat, Turkey

**Keywords:** Smoking cessation, Primary care, Artificial intelligence, Patient education, Readability, DISCERN, Digital health

## Abstract

**Background:**

Artificial intelligence (AI) has emerged as a promising tool to support smoking cessation in primary care, particularly for populations underserved by traditional interventions. However, the quality of AI-generated smoking cessation advice remains understudied, especially in low-resource settings and among vulnerable groups such as adolescents.

**Objective:**

This study aims to evaluate AI-generated responses to smoking cessation questions for patient education in primary care, comparing different AI programs in terms of knowledge, readability, and quality.

**Methods:**

Ten publicly accessible AI programs were prompted in Turkish with 24 standardized, open-ended smoking cessation questions framed as a patient consultation. Two family medicine specialists independently assessed each response’s readability using Ateşman’s Readability Index, reliability using the DISCERN instrument, and accuracy and motivational interviewing quality using a bespoke rubric and OARS (Open questions, Affirmations, Reflections, Summaries) framework. Inter‐rater agreement was evaluated via intraclass correlation. Descriptive statistics were computed for readability scores, DISCERN ratings, and accuracy grades.

**Results:**

All AI programs provided at least partially correct answers to all questions. The average readability score was 54.90 (medium difficulty) according to Atesman’s Index. The mean DISCERN score was 66 ± 5.2, indicating excellent quality. Three AI programs incorporated core motivational interviewing skills. The most accurately answered question concerned e-cigarettes’ harm compared to traditional cigarettes, while medication advice was least evidence-based.

**Conclusions:**

Free AI chatbots deliver reliably accurate and moderately readable smoking cessation advice, supporting their potential role as patient education adjuncts in primary care—particularly for individuals with at least a high school education. Further research should compare AI-assisted versus clinician‐led interventions on smoking cessation outcomes.

**Supplementary Information:**

The online version contains supplementary material available at 10.1186/s12875-026-03360-z.

## Introduction

All healthcare professionals can play a role in educating patients and providing brief advice on smoking cessation [1]. However, these interventions are underutilised due to inadequate clinician training and organisational support [2]. From 1990 to 2021, 4.30 billion lives were lost because of smoking as a risk factor of mortality. The Elimination-2023 scenario quantifies the maximum potential future health benefits; by 2050, it is expected to prevent 2 billion deaths by reducing global smoking by 5% [3].

Recent evidence suggests that primary care settings present an important opportunity for smoking cessation. Several studies have examined the impact of various interventions, from simple advice to comprehensive assistance in primary care. However, there are limitations and barriers in primary care regarding smoking cessation [4]. A lack of time and organisational problems were identified as major obstacles by research conducted among Spanish family physicians [5]. With similar concerns, Nordic family physicians believe that smoking cessation takes too much time due to low quitting rates [6]. Motivational interviewing, Level B evidence for smoking cessation, is also time-consuming [7].

In particular, inadequate follow-up between appointments creates a critical care gap during the high-risk early abstinence phase. Since smoking cessation is a chronic, relapsing condition that requires ongoing behavioural support, the period between primary care visits is a vulnerable time. Digital health tools—especially AI-supported conversational systems—can act as structured adjuncts that extend evidence-based care beyond the consultation.

Existing research acknowledges the vital role played by the internet among individuals. The internet is a dependable source that influences individuals’ actions concerning their health [8, 9]. Also, they increasingly use artificial intelligence to gather medical information and make health-related decisions. Artificial intelligence can enhance these decision-making processes and generate solutions more effectively and tailored [10, 11].

Not only commonly used by patients, health services have also used artificial intelligence in order to improve diagnostic processes, especially in radiology, pathology, dermatology, and ophthalmology [11]. There are studies evaluating artificial intelligence’s responses to patients’ frequently asked questions on various topics, such as “thyroid nodules”, “obstructive apnea”, and “atrial fibrillation” [12–14]. The fact that machine learning can provide family physicians with patient education materials for smoking cessation remains largely unknown.

**Fig. 1:**
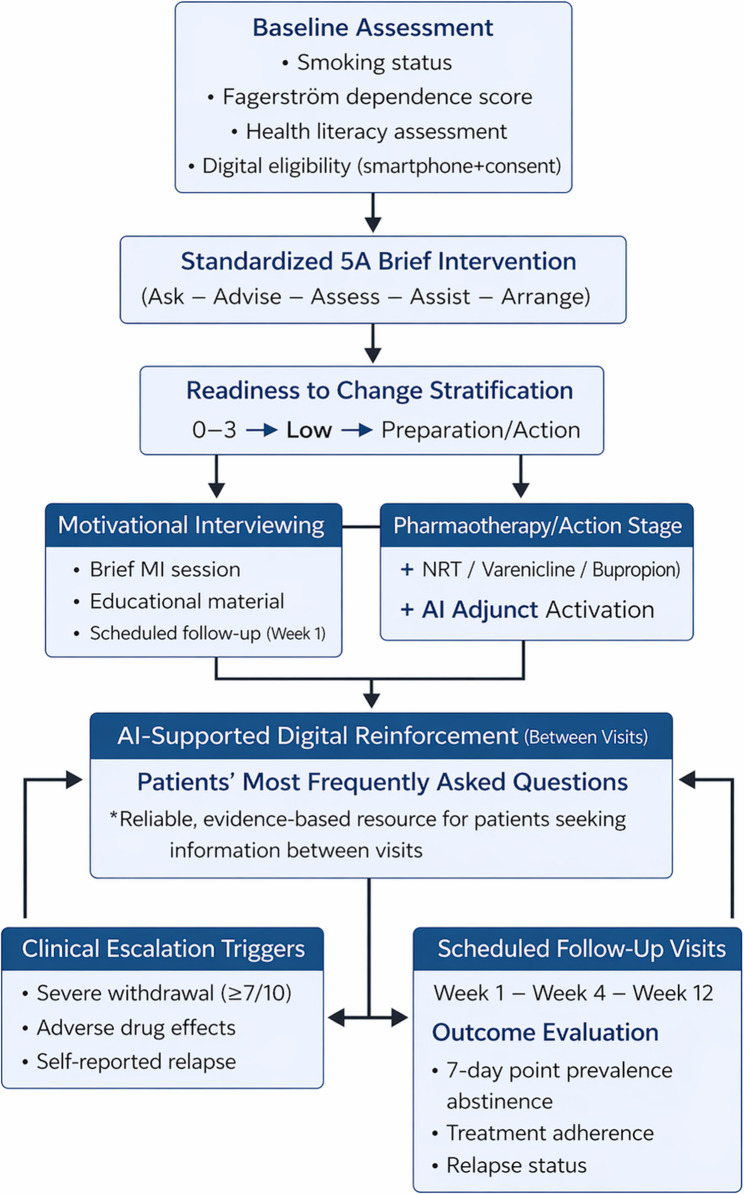
AI-Integrated Smoking Cessation Framework

This study examines whether AI chatbots provide medically accurate information to individuals trying to quit smoking, especially during the period between doctor visits, when access to direct consultation is limited (Fig. 1). The assessment focused on AI chatbot responses to common questions about smoking cessation within a primary care setting, specifically evaluating readability, reliability and information quality, medical accuracy, country relevance, and motivational interviewing communication skills.

## Methods

### Study design and sampling

This study was designed as a descriptive, cross-sectional assessment of artificial intelligence (AI)-generated responses related to smoking cessation. AI programs that were publicly accessible and capable of providing health-related information were identified through a structured web search carried out between April and May 2025. The search process included commonly used search engines and AI tools to compile a comprehensive list of available systems.

Only AI programmes designed to produce informational responses were included. Tools mainly created for other goals (e.g., video generation or presentation design) were excluded. At the time of the study, 12 eligible AI programmes were identified. Two were excluded: one requiring mandatory membership before response generation and another not accessible in Türkiye. As a result, 10 AI programmes with freely available versions were included.

The inclusion of openly accessible AI tools was deliberate to mirror real-world patient behaviour, as individuals typically use open-access platforms without assistance. Although these tools are publicly available, the study was conducted within a primary care (PHC) context because the researchers are family physicians aiming to assess how AI-generated information might influence daily clinical practice, patient expectations, and physician–patient interactions.

### Question development

The questions were developed by two researchers who are family medicine specialists with Master’s degrees in medical education. An initial pool of questions was created based on the most frequently addressed topics in smoking cessation identified from UpToDate and the Turkish Ministry of Health’s official health information platform. These sources were chosen because they align with evidence-based clinical guidelines and routine primary care practice.

A structured discussion was conducted among the researchers to refine the question set. Repetitive questions were eliminated, and similar items were combined. Furthermore, three new questions reflecting common patient concerns in clinical practice were added, even though they were not explicitly listed in the source materials. No strict upper limit on the number of questions was set to ensure comprehensive coverage.

A pilot evaluation was carried out with five actively smoking individuals to assess the relevance and completeness of the questions. Participants were asked whether additional questions should be included. Although two participants suggested adding questions about the perceived benefits of smoking or tobacco use, these were excluded to keep alignment with the study population—people who have decided to quit smoking and are in the preparation or action stage.

A total of 24 open-ended questions were finalised. All questions were asked in Turkish to ensure ecological validity, and responses were collected in Turkish. The English translations of the questions are presented in Table 1.

**Table 1:** Assesment questions

Q1	What should I do first to quit smoking?
Q2	What are the benefits of quitting smoking?
Q3	Will I gain weight after quitting smoking?
Q4	What symptoms might I experience after quitting smoking?
Q5	I’ve tried to quit smoking many times but haven’t been successful. What should I do?
Q6	How can my family physician help me during the smoking cessation process?
Q7	Should I use medication to quit smoking?
Q8	Is smoking during pregnancy harmful to my baby?
Q9	What can I do to increase my chances of successfully quitting smoking?
Q10	How can I help a loved one quit smoking?
Q11	How long after quitting am I considered to have completely quit?
Q12	What is found in cigarette smoke?
Q13	Are light cigarettes less harmful to health?
Q14	Are light and flavored cigarettes less harmful to health?
Q15	What is secondhand smoke?
Q16	What happens if I smoke in a place where it’s legally prohibited?
Q17	Is it too late for me to quit smoking?
Q18	Is hookah (waterpipe) less harmful than cigarettes?
Q19	Is electronic cigarette less harmful than regular cigarettes?
Q20	Are electronic cigarettes effective in helping people quit smoking?
Q21	Are complementary and alternative therapies effective for smoking cessation?
Q22	Is it impossible to quit smoking without first mentally committing to it?
Q23	I only smoke socially or while drinking coffee; I don’t carry a pack. Am I still addicted?
Q24	Is there a national helpline I can call for support after deciding to quit smoking?

### Data collection

To standardise AI responses and mimic a real-world consultation setting, all researchers collaboratively developed a unified prompt: “I live in Turkey. I decided to quit smoking. I went to my family physician, and he made suggestions and said that I could get answers by asking you my questions. My question is…”.

Each researcher started a new session for every AI program to reduce bias from previous context. All 24 questions were entered into each AI system, and responses were recorded exactly as given in a structured document (Table 1). 

### Evaluation of responses from AI tools

The researchers’ main responsibility was to assess the responses using a rubric-based method across four specified areas: readability, reliability, accuracy, and communication abilities. These areas were chosen to represent essential aspects of patient education in primary care, including understandability, trustworthiness, clinical correctness, and capacity for behavioural support.

The rubric used to assess accuracy and communication was created by the researchers based on existing literature on health information quality, patient education, and patient-centered communication. Relevant frameworks, including principles of evidence-based medicine and primary care communication models, were reviewed during its development.

Rubrics are structured evaluation tools widely used to assess specific performances or outcomes in educational and research settings, providing transparent and reproducible scoring criteria [15, 16]. The rubric was repeatedly refined through structured discussions among the research team until consensus was reached on the evaluation criteria. It included clearly defined scoring categories to assess both the medical accuracy of the information and the quality of communication.

In this study’s context, accuracy refers to the fundamental medical correctness and clinical suitability of the responses. It was assessed using a 5-point Likert-type scale: incorrect, partially correct, correct, correct with either a statistic or citation (“correct+”), and correct with both a statistic and citation (“perfect”) [13].

The readability of the responses was assessed by Ateşman’s Readability Index [198,825-(40,175xnumber of syllables/number of words) - (2,610xnumber of words/ number of sentences)]. The Ateşman Readability Formula was developed by Ateşman in 1992 for Turkish to measure how easily a text can be understood by readers. The most used variables in measuring readability are average sentence length and average word length. According to the Ateşman formula, the readability score is interpreted as follows: 1–29 very difficult, 30–49 difficult, 50–69 medium difficulty, 70–89 easy, and 90–100 very easy [17].

The reliability of the content was measured by Quality Criteria for Consumer Health Information (DISCERN). The DISCERN scale was developed by Charnock and started to be used in the health field in 1998. It was translated into Turkish by Gökdoğan et al. DISCERN consists of 16 questions and is graded from 1 to 5. A grade of 1 indicates the video is bad; a grade of 5 indicates it is very good. It also consists of three sections: the first 8 questions evaluate reliability, questions 9–15 evaluate the quality of information for treatment, and the last question evaluates general information. According to this tool, AI responses are divided into 5 groups by taking into account the total average scores: 16–26 is considered very bad, 27–38 is considered weak, 39–50 is considered medium, 51–62 is considered good, and a score above 63 is considered excellent [18].

Communication skills were evaluated based on clarity, coherence, empathy, and the ability to support behaviour change in accordance with primary care principles. Additionally, responses were assessed using motivational interviewing–aligned smoking-cessation strategies based on the 5 A framework (Ask, Advise, Assess, Assist, Arrange) [7]. Specifically, responses were scrutinised for their capacity to identify smoking status (Ask), offer clear and personalised advice to quit (Advise), evaluate readiness to quit (Assess), provide practical support and strategies (Assist), and recommend follow-up or continuity of care (Arrange).

All responses were independently assessed by two researchers. Disagreements in scoring were discussed and resolved through consensus. Before the main analysis, a pilot evaluation was performed on a subset of responses to ensure consistency in scoring and to calibrate the evaluators.

The normality of continuous variables was evaluated using both visual methods (histograms and probability plots) and statistical tests (Kolmogorov-Smirnov/Shapiro-Wilk tests). Descriptive statistics were reported as frequencies, percentages, mean ± standard deviation, and median (min-max).

This study was conducted using publicly available, anonymised data obtained from AI chatbots and did not involve any direct interaction with human participants. Therefore, no informed consent was required. The study protocol was reviewed and approved by the Bursa Uludağ University Medical School Non-Clinical Ethical Research Committee, under the approval number [2025/4 − 3].

The research was carried out in accordance with the ethical standards of the institutional ethics committee and with the principles of the Declaration of Helsinki.

## Results

Out of 24 open-ended questions, there was no AI program that failed to understand or was unable to answer them. All questions received at least a “partially correct” response. The assessment questions are presented in Table 2 for all AI programmes. Of all responses to the artificial programmes, the most evidence-based question answered was whether electronic cigarettes were less harmful than traditional cigarettes. The least evidence-based answered question was “Shall I use medication to quit smoking?”

**Table 2:**
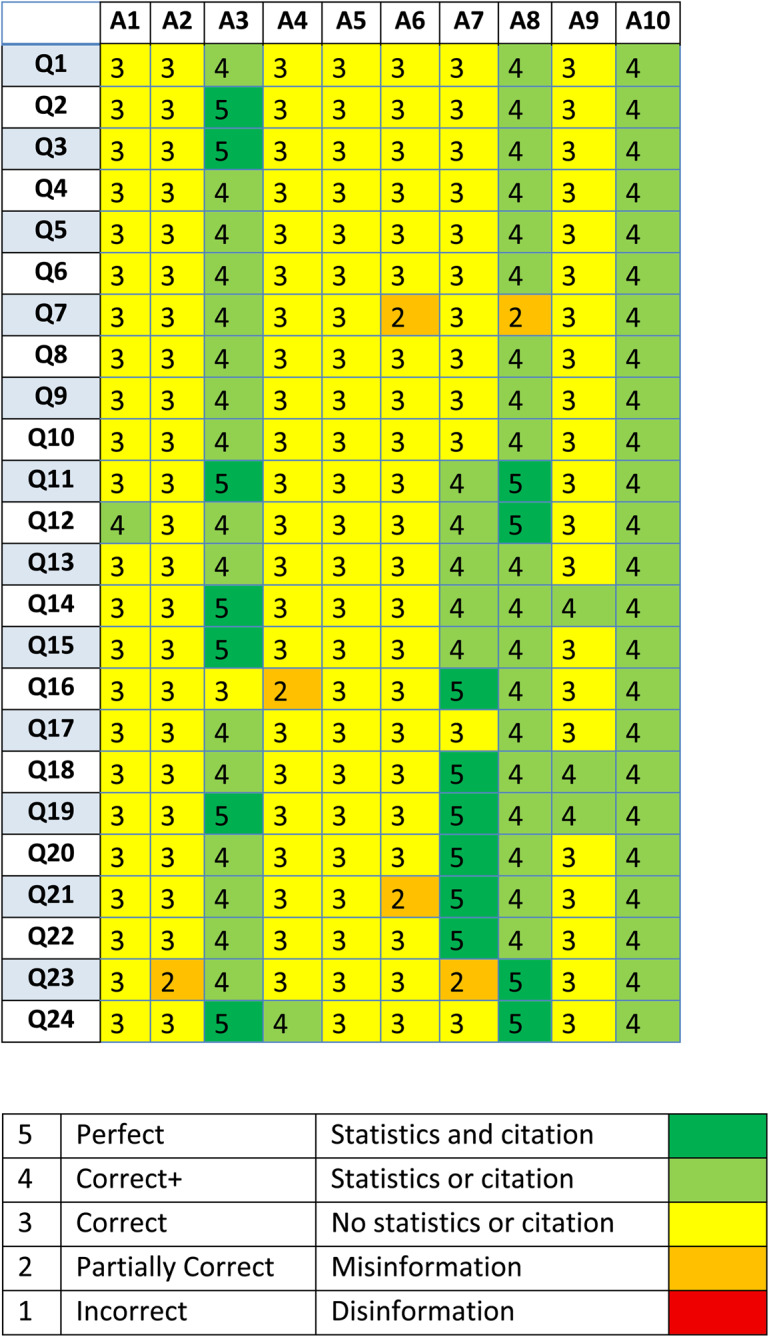
Comparative Analysis of AI Responses to Smoking Cessation Questions

### Readability

The overall mean Ateşman readability score was 54.90 (range 36.46–65.20), indicating medium difficulty. Eight platforms produced text within the medium range, while two produced responses classified as difficult. None achieved readability levels described as “easy” or “very easy.”

### Information quality (DISCERN)

The average DISCERN score was 66 ± 5.2 (range 58–75), indicating a label of “excellent” quality. Differences between platforms were not statistically significant (*p* = 0.25). The internal consistency of the DISCERN tool was high (Cronbach’s α = 0.91; standardised α = 0.93).

Inter-rater reliability for DISCERN was measured with a two-way random-effects intraclass correlation coefficient (absolute agreement model). The single-measure ICC was 0.84 (95% CI 0.71–0.96; *p* < 0.001).

### Accuracy

**Fig. 2:**
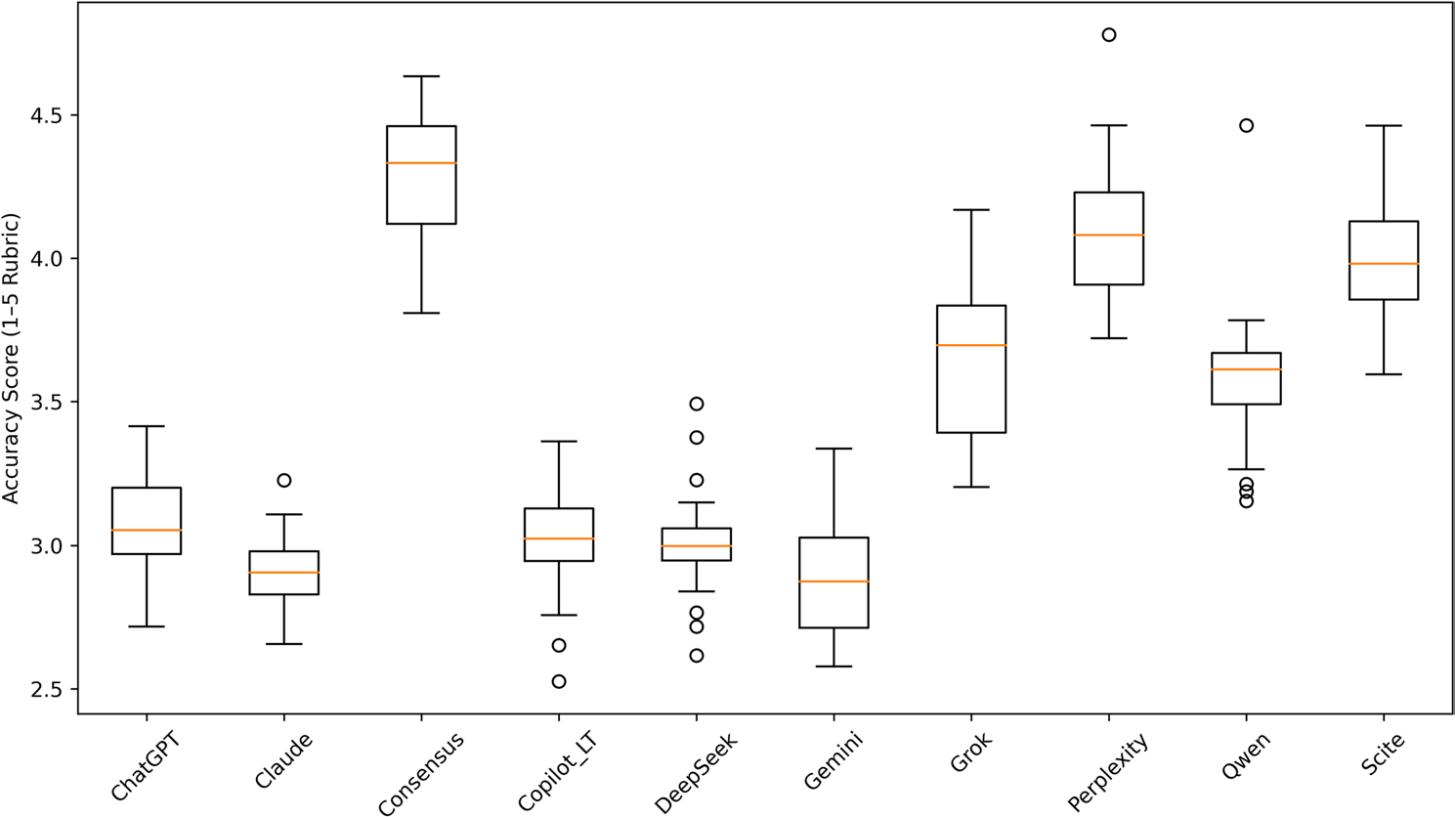
Distribution of Accuracy Scores Across Al Platforms

The internal consistency of the 24-question accuracy rubric was excellent (Cronbach’s α = 0.975; standardised α = 0.980). Accuracy differed significantly across platforms (Friedman χ² [9] = 155.92, *p* < 0.001), with a large effect size (Kendall’s W = 0.72). Mean accuracy scores ranged from 2.92 to 4.25, showing notable variability. Platforms with higher scores more often used statistical framing or cited evidence sources. Conversely, lower-scoring platforms often gave general advice without clear evidence markers. The most reliably accurate topic concerned the comparative harms of electronic versus combustible cigarettes. Responses related to pharmacotherapy were less consistently marked with evidence, with frequent omission of cytisine. Variability and central tendency of model accuracy are displayed using boxplot distributions to clearly compare performance variation (Fig. 2).

### Content-level analysis

It is revealed that systematic patterns of omission and contextual deviation exist. Questions related to pharmacotherapy showed the highest frequency of clinically relevant omissions, especially concerning cytisine, a WHO-recommended and nationally available treatment. Several platforms offered general reassurance about medication safety without addressing contraindications or patient-specific factors. In certain cases, alternative therapies were described in a favourable manner despite limited high-quality evidence. No outright fabrication of pharmacological agents was observed; however, overgeneralised framing and incomplete contextualisation were present (Table 2).

### Communication content analysis

Motivational Interviewing competence is assessed using rubrics. The rubrics focus on core skills of motivational interviewing, abbreviated as OARS (open questions, affirmation, reflection, and summarising). The most surprising aspect of the data is that three out of ten artificial intelligence responses employ exactly the same core skill of OARS.

## Discussion

Previous studies have highlighted the significance of artificial intelligence in medicine [19]. Several reports have demonstrated that ChatGPT is understandable and dependable for certain information related to patient empowerment [12–14, 19]. In reviewing the literature, no data were identified on the use of AI for smoking cessation as a patient education tool in primary care. Very little has been published on comparing different artificial intelligence programmes concerning neurological issues [20]. In this context, this may be one of the first studies in the literature to compare the quality of information from AI bots used in patient education for smoking cessation.

From all the responses to artificial programs, the most evidence-based question answered was whether electronic cigarettes were less harmful than traditional cigarettes. This could indicate that artificial programs capture society’s popular subjects.

The least evidence-based answered question was “Shall I use medication to quit smoking?” All responses began with general treatments for smoking cessation, followed by advice to consult a family doctor or other medical professional, and then provided information on medications. However, none of the artificial programmes included information about cytisine. Cytisine is a medication for smoking cessation according to the WHO Clinical Treatment Guideline for Tobacco Cessation in Adults, 2024 [21]. It binds to nicotinic acetylcholine receptors (nAChRs) in the brain as a partial agonist and is available free of charge through the Turkish National Health Insurance but lacks FDA approval [22]. This may be a cause for blinding the Al bots among correct medication information.

In a study examining reproductive health information provided by artificial intelligence, the average Flesh-Kincaid score for ChatGPT responses was 13.1, corresponding to college-level reading ability [23]. In this study, we posed the questions in Turkish. Instead of the Flesh-Kincaid Score, we employed the Ateşman readability index, yielding an average score of 54.9, indicating a moderate level of readability and suggesting that the content is appropriate for individuals with at least a high school education [24]. This implies that all artificial intelligence programmes, including free versions, can be read and understood by individuals with at least a high school education in relation to quitting smoking; at the same age as adolescents and older.

The reliability of answers was assessed using the DISCERN score. The average DISCERN scores for texts generated by artificial intelligence programmes were 66 ± 5.2, which equates to 4.8. The average scores for ChatGPT and Gemini were 3.8 for common neurological disorders [20]. In another study of ChatGPT’s responses to patients with hip replacements, orthopaedists rated the reliability at 4.6 [25]. Comparing these findings with other studies confirms that all artificial programmes, even the free versions, are reliable for smoking cessation and other health scenarios.

One unexpected finding was that three out of ten responses from artificial intelligence shared the same core skill: OARS, indicating potential for future applications [26]. Additionally, it enhances the case for recommending it as an outpatient tool for smoking cessation in primary care.

## Limitations

The evaluation was performed at a single point in time and was limited to outputs in Turkish. As AI models develop quickly, their performance may vary over time. The rubric-based assessment focused on content quality rather than clinical effectiveness, and no behavioural outcomes were recorded.

## Conclusions

The main aim of this study was to evaluate the quality of artificial intelligence responses on smoking cessation for patient education in primary care. The most obvious finding from this study is that it is more reliable and easier to read than responses to other health issues. This indicates that artificial intelligence has significant potential for self-learning and development in an academic context. Overall, these results suggest that all of these intelligence programmes could be recommended for patient education in primary care, especially for those seeking help with smoking cessation who have a high school level education or higher. The findings will also be of interest to family physicians, as they could save time and effort in primary care smoking-cessation interventions. Future research should compare the smoking rates of patients assisted by AI with those assisted by family physicians.

## Supplementary Information

Supplementary Material 1.

## Data Availability

All data supporting the findings of this study are available within the paper and its Supplementary Information. For further information, the corresponding author will share data by e-mail.

## Abbreviations

AI: Artificial Intelligence
