# Supplementary material for: Assessing AI-generated smoking cessation advice for patient education in primary care
Source: BMC Prim Care. 2026 May 18;27:206. doi: 10.1186/s12875-026-03360-z (PMC13198041; doi:10.1186/s12875-026-03360-z)
Supplement: Supplementary file 1 — Supplementary Material 1. [file 12875_2026_3360_MOESM1_ESM.docx]

| A1 | CHATGPT |
| --- | --- |
| A2 | CLAUDE |
| A3 | CONSENSUS |
| A4 | COPILOT |
| A5 | DEEPSEEK |
| A6 | GEMİNİ |
| A7 | GROK |
| A8 | PERLEXITY |
| A9 | QWEN |
| A10 | SCITE |
